# Targeting 2-Oxoglutarate–Dependent Dioxygenases Promotes Metabolic Reprogramming That Protects against Lethal SARS-CoV-2 Infection in the K18-hACE2 Transgenic Mouse Model

**DOI:** 10.4049/immunohorizons.2300048

**Published:** 2023-07-07

**Authors:** Forrest Jessop, Benjamin Schwarz, Eric Bohrnsen, Molly Miltko, Carl Shaia, Catharine M. Bosio

**Affiliations:** * Immunity to Pulmonary Pathogens Section, Laboratory of Bacteriology, Rocky Mountain Laboratories, National Institute of Allergy and Infectious Disease, Hamilton, MT; † Rocky Mountain Veterinary Branch, Rocky Mountain Laboratories, National Institute of Allergy and Infectious Disease, Hamilton, MT

## Abstract

Dysregulation of host metabolism is a feature of lethal SARS-CoV-2 infection. Perturbations in α-ketoglutarate levels can elicit metabolic reprogramming through 2-oxoglutarate–dependent dioxygenases (2-ODDGs), leading to stabilization of the transcription factor HIF-1α. HIF1-α activation has been reported to promote antiviral mechanisms against SARS-CoV-2 through direct regulation of ACE2 expression (a receptor required for viral entry). However, given the numerous pathways HIF-1α serves to regulate it is possible that there are other undefined metabolic mechanisms contributing to the pathogenesis of SARS-CoV-2 independent of ACE2 downregulation. In this study, we used in vitro and in vivo models in which HIF-1α modulation of ACE2 expression was negated, allowing for isolated characterization of the host metabolic response within SARS-CoV-2 disease pathogenesis. We demonstrated that SARS-CoV-2 infection limited stabilization of HIF-1α and associated mitochondrial metabolic reprogramming by maintaining activity of the 2-ODDG prolyl hydroxylases. Inhibition of 2-ODDGs with dimethyloxalylglycine promoted HIF-1α stabilization following SARS-CoV-2 infection, and significantly increased survival among SARS-CoV-2–infected mice compared with vehicle controls. However, unlike previous reports, the mechanism by which activation of HIF-1α responses contributed to survival was not through impairment of viral replication. Rather, dimethyloxalylglycine treatment facilitated direct effects on host metabolism including increased glycolysis and resolution of dysregulated pools of metabolites, which correlated with reduced morbidity. Taken together, these data identify (to our knowledge) a novel function of α-ketoglutarate–sensing platforms, including those responsible for HIF-1α stabilization, in the resolution of SARS-CoV-2 infection and support targeting these metabolic nodes as a viable therapeutic strategy to limit disease severity during infection.

## Introduction

Respiratory failure resulting from SARS-CoV-2 infection is associated with high mortality rates and can lead to persistent sequelae in survivors (1). The etiology of respiratory failure following SARS-CoV-2 infection shares many pathological idiosyncrasies with other infectious and noninfectious lung injuries that culminate in acute respiratory distress syndrome (ARDS). As an endpoint outcome of infection, ARDS is inherently difficult to treat. Therefore, identification of host factors that predispose one to ARDS is needed to develop effective interventions prior to the culmination of catastrophic pathology. Only a minority of SARS-CoV-2 infections progress to ARDS, which occurs predominantly in individuals with dysregulated immune and/or metabolic comorbidities (2). Accordingly, examination of pathogenesis within highly susceptible animal models, such as the B6.Cg-Tg(K18-ACE2)2Prlmn/J (K18-hACE2) mouse, may assist in identification of host factors that contribute to increased morbidity and mortality rates in susceptible populations.

Lethal murine models of SARS-CoV-2 infection have several distinct phases of infection. First, there is a period of viral replication during which inflammatory and supportive metabolic reprogramming are absent (3). After peak viral burdens have been reached, this quiescent period is followed by tissue-damaging inflammation triggered by host danger-associated molecular patterns (3). At later stages of severe SARS-CoV-2 infection, hypoxia and hypoxemia occur, indicative of damage to the alveolar/capillary interface and dysregulation of the pulmonary vasculature through thrombosis or vasoconstriction, which correlate with poor outcomes in SARS-CoV-2–infected individuals (4, 5). The absence of these signatures prior to the point of peak viremia in animal models of infection suggests that the late-stage metabolic responses may be overcompensating for an inadequate early response. Moreover, these findings suggest that absence of an early host response is likely due to viral evasion of immune recognition and/or a suppression of metabolic platforms required for innate immune activation. Accordingly, therapeutically targeting metabolic dysregulation to support immune functions in the host may be a viable approach in established SARS-CoV-2 infection (6, 7).

Targeting 2-oxoglutarate–dependent dioxygenases (2-OGDDs) may have therapeutic potential for limiting lethal SARS-CoV-2 infections, as these enzymes sense and respond to perturbations in α-ketoglutarate levels associated with dysregulation in central metabolism. α-Ketoglutarate is a tricarboxylic acid cycle intermediate predominantly generated via glutamine metabolism that is continually sensed by 2-OGDD, along with other cofactors including oxygen, iron, and ascorbate (8). Decreases in intracellular α-ketoglutarate levels lead to reduced 2-OGDD activity, which in turn promotes adaptive programs ranging from retailoring central metabolism to epigenetic modifications (9). The most predominant 2-OGDD involved in the metabolic response to infection are prolyl hydroxylases (PHDs), which under homeostatic levels of α-ketoglutarate and oxygen promote ubiquitination and targeted degradation of HIF-1α (8). During metabolic stress where PHD substrates and cofactors are limited by reduced mitochondrial respiration, HIF-1α accumulates and initiates critical metabolic adaptations including upregulation of glycolysis to compensate for reduced mitochondrial bioenergetics (10, 11). Stabilization of HIF-1α stabilization can also engage pathways that limit mitochondrial reactive oxygen species (ROS) production (10, 11). Furthermore, HIF-1α–dependent upregulation of glycolysis also facilitates proinflammatory metabolic pathways needed to support cytokine production (11). Many of the enzymes increased by HIF-1α within the glycolytic pathway possess moonlighting functions essential for production of proinflammatory and antiviral effectors (12). Increased HIF-1α levels have been reported in cardiovascular tissue regions that display less cellular damage postmortem in COVID-19 patients, suggesting that HIF-1α activation was correlated with tissue protection (13). As described above, early postinfection, SARS-CoV-2 evades triggering-increased glycolysis until after peak viral load is achieved (3), suggesting the host’s ability to sense perturbations in α-ketoglutarate metabolism and/or HIF-1α stabilization may be limited or manipulated. Therefore, timely targeting of the α-ketoglutarate–sensing pathways to upregulate of HIF-1α responses may interrupt early metabolic programs required for SARS-CoV-2 disease pathogenesis by supporting essential proinflammatory and cellular viability functions.

Recent reports have demonstrated that stabilization of HIF-1α via inhibition of the PHD contributed to control of SARS-CoV-2 infection in vitro and in a nonlethal hamster model (14, 15). In those studies, the primary mechanism by which HIF-1α stabilization contributed to the outcome of infection was via downregulation of the receptors required for viral entry (ACE2 and TMPRSS2), effectively limiting viral burdens. However, as stated above, PHD and HIF-1α are central regulators of host metabolic responses and their stabilization may affect host pathways that contribute differently to survival of SARS-CoV-2 than those previously described.

In this study, we used in vitro and in vivo models where PHD/HIF-mediated regulation of ACE2 expression was dissociated to allow for identification of other prosurvival host responses engaged by α-ketoglutarate–sensing pathways during SARS-CoV-2 infection. We demonstrate that SARS-CoV-2 infection limited HIF-1α stabilization and the resultant prosurvival metabolic reprogramming during the early phase of infection. Treatment with a competitive inhibitor of 2-ODGGs, the α-ketoglutarate-derived dimethyloxalylglycine (DMOG), to stabilize HIF-1α improved survival of infected K18-hACE2 mice without affecting hACE2 expression. By extension, survival was not correlated with changes in viral replication/burden. Rather, enhanced host glycolysis, increased inflammatory mediators, reversal of dysregulated amino acids pools, and reversal of loss of hypoxanthine all correlated with protection. Taken together, these data demonstrate that there are multiple mechanisms by which limiting 2-OGDD activity contributes to survival of SARS-CoV-2, and targeting the associated host metabolic responses may be an effective intervention strategy.

## Materials and Methods

### SARS-CoV-2

SARS-CoV-2 (USA/WA1/2020) was obtained from BEI Resources and propagated in Vero cells to generate viral stocks. Stock solutions were frozen at –80°C until use.

### In vitro infection

A549 cells engineered to express hACE2 were donated by Dr. Paul Beare at the Rocky Mountain Laboratories. A detailed description of the generation of the A549 cells has been reported elsewhere (16). A549 cells were plated at 2 × 10^4^ cells per well in a 48-well tissue culture plate in complete DMEM (cDMEM; glucose, HEPES, l-glutamine, nonessential amino acids, sodium pyruvate [all from Thermo Fisher Scientific], and 10% heat-inactivated FBS [Atlas Biologicals]). After 24 h, medium was removed, and SARS-CoV-2 inoculum was added to the cells at a multiplicity of infection (MOI) of 0.01. Virus was allowed to infect the cells for 1 h at 37°C with 5% CO_2_. Inoculum was removed and fresh cDMEM was added back to the A549 cultures. After 1 h, DMOG (Cayman Chemical) was added to the culture at the indicated concentrations. Viral titer, HIF-α stabilization, and cell death were assessed where indicated.

### Extracellular flux analysis

A549 cells were plated at 1 × 10^4^ cells per well in a 96-well tissue culture plate in cDMEM. After 24 h, medium was removed, and SARS-CoV-2 inoculum was added to the cells at an MOI of 0.01. Virus was allowed to infect the cells for 1 h at 37°C with 5% CO_2_. Inoculum was removed and fresh cDMEM was added back to the A549 cultures. After 1 h, DMOG (Cayman Chemical) was added to the culture. Extracellular flux analysis was performed using a Seahorse XFe96 (Agilent Technologies) at 10, 24, and/or 48 h postinfection where indicated. For assessment of mitochondrial function, we performed a mitochondrial stress test as previously described (17). Briefly, mitochondrial function was assessed in minimal medium (minimal DMEM containing sodium pyruvate, l-glutamine, and glucose [Agilent Technologies]) in the context of oligomycin (2 µM), FCCP (2 µM), and rotenone/antimycin (2 µM) exposure. Basal Respiration, ATP-dependent oxygen consumption rate (OCR), proton leak, maximal respiration, and nonmitochondrial respiration were calculated according to the manufacturer’s instructions. For assessment of glycolytic function, we performed a glycolysis stress test in minimal medium (minimal DMEM containing only l-glutamine [Agilent Technologies]) and calculated glycolysis-associated extracellular acidification rate after injection of glucose (10 mM), oligomycin (2 µM), or 2-deoxyglucose (50 mM).

### Quantification of cell death

A549 cells were stained using NucGreen Dead 488 ReadyProbes reagent (Thermo Fisher Scientific) dead indicator, which stains cells that have lost plasma membrane integrity. Cell nuclei were counterstained using DAPI (Thermo Fisher Scientific). Cell cultures were imaged 72 h after SARS-CoV-2 infection using an EVOS FL Auto 2 microscope (Thermo Fisher Scientific). Cell death was quantified by enumerating the number of NucGreen-positive cells proportional to total cell DAPI-stained nuclei per field of view (17). Image analysis was performed using ImageJ. Additionally, at 72 h postinfection, extracellular lactate dehydrogenase (LDH) activity was assessed using the CytoTox 96 nonradioactive cytotoxicity assay (Promega) according to the manufacturer’s instructions.

### Mice

K18-hACE2 mice of mixed sexes were purchased from The Jackson Laboratory or bred in-house. All mice were specific pathogen-free and housed in animal biosafety level 3 facilities at Rocky Mountain Laboratories. K18-hACE2 mice were used between 8 and 12 wk of age, housed on a 12-h light/12-h dark cycle, and provided food and water ad libitum. To establish infection, mice were anesthetized with ketamine/xylazine, and 25 μl of SARS-CoV-2 (10^2^ 50% tissue culture-infective dose [TCID_50_]) inoculum diluted in PBS was delivered by intranasal instillation in the left nare. Mice were treated with DMOG 24 h prior to infection, 8 h postinfection, and daily thereafter up to 12 d postinfection. DMOG (500 mg/kg) was administered i.p. in 200 μl. The dose of 500 mg/kg DMOG was selected based the lowest dose that elicited consistent changes in metabolic intermediates of glycolysis in the lung 24 h after a bolus dose (Supplemental Fig. 2A). Survival was monitored out to 20 d postinfection, and mice were humanely euthanized when defined clinical signs of infection were reached. For kinetic studies, mice were anesthetized with ketamine/xylazine and infected with SARS-CoV-2 as described above or instilled with PBS as mock-infected controls. Mice were treated with DMOG as described above or vehicle only. On days 3 and 5 postinfection, mice were euthanized and lungs were excised for analysis of metabolites, inflammatory cytokines/chemokines, and/or pathology. All studies involving K18-hACE2 mice were performed with the approval of and in accordance with the Animal Care and Use Committee at Rocky Mountain Laboratories (animal study protocol 2020-29-E).

### Histology

Lungs were inflated with 1 ml of PBS, removed, and fixed in 10% neutral-buffered formalin with two changes of formalin for a minimum of 7 d prior to processing. Lung tissues were placed in cassettes and processed with a Tissue-Tek VIP 6 tissue processor (Sakura Finetek) on a 12-h automated schedule using a graded series of ethanol, xylene, and PureAffin (Cancer Diagnostics). Paraffin-embedded tissues were sectioned at 5-μm thickness and dried overnight at 42°C before staining. Fixed tissue sections were stained with H&E, after which stained slides were examined on an Olympus BX53 light microscope equipped with an Olympus DP74 camera and associated cellSens Dimension 1.4.1 software. Pathological analysis was performed blinded by a board-certified pathologist. Lesions were scored from 0 (no lesions) to 5 (severe) for vascular reaction with perivascular inflammation or septal thickening with interstitial inflammation as previously described (3).

### Tissue processing for cytokine and viral burden quantification

Lungs were aseptically removed and placed in cold complete DMEM. Tissues were homogenized by grinding through a sterile S/S type 304 no. 60 wire mesh screen (Belleville Wire Cloth) using a 5-ml syringe plunger. Tissues were then centrifuged at 9400 × *g* for 5 min, and supernatant was collected and frozen at –80°C until downstream analysis. Murine serum was obtained from isolated blood without anticoagulation treatment following a 30-min incubation at room temperature and then centrifugation at 1300 × *g* for 15 min. Serum was frozen at –80°C until downstream analysis.

### Quantification of viral burden by TCID_50_ assay

Vero cells (CCL-81, American Type Culture Collection) were plated at 1 × 10^4^ per well in a flat-bottom 96-well plate in cDMEM. After 24 h, Vero cells were overlaid with cell supernatants isolated from infected A549 cells or homogenized lung tissue samples that had been serially diluted in cDMEM. Serially diluted samples were added to the Vero cultures and then incubated for 1 h at 37°C with 5% CO_2_. Samples were removed and fresh cDMEM was added to the cells. Cells were incubated for 72 h and evaluated for cellular lysis/well clearance. Briefly, supernatant was removed and cells were fixed with 100 μl of 4% formalin for 1 h at room temperature. Formalin was removed and cells were washed once with PBS, followed by addition of 25 μl of 0.1% crystal violet stain in methanol to each well. Cells were incubated for an additional 15 min, after which the stain was removed and cells were washed twice with distilled water. TCID_50_ was calculated according to the Reed–Muench method.

### Inflammatory cytokine, HIF-1α, and ACE2 protein quantification

Inflammatory cytokines/chemokines were quantified in whole-lung homogenates using a Meso Scale multiplex cytokine array following the manufacturer’s instructions (U-PLEX Biomarker Group 1, Meso Scale Diagnostics). HIF-1α was quantified using ELISA (R&D Systems) and confirmed by Western blot using the ProteinSimple Wes microcapillary system (Bio-Techne) following the manufacturer’s instructions using an anti–HIF-1α Ab (D1S7W, catalog no. 36169S, Cell Signaling Technology). ACE2 was quantified in A549 cell lysates (using anti–ACE-2 Ab, catalog no. 4355S, Cell Signaling Technology) using a ProteinSimple Wes microcapillary Western blot. Total protein levels were determined using the total protein detection module for chemiluminescence assays for Simple Western according to the manufacturer’s instructions (Bio-Techne). The target protein signal was normalized to total protein levels.

### Quantification of hACE2 expression by quantitative reverse trasnscription PCR

RNA was isolated from lung tissue in RLT buffer using a QIAamp RNeasy mini kit according to the manufacturer’s instructions (Qiagen). cDNA was generated from RNA isolates using a SuperScript VILO cDNA synthesis kit according to the manufacturer’s instructions (Invitrogen). hACE2 was quantified by quantitative reverse trasnscription PCR using the following primers: forward, 5′-TGGAAAATCARAACCCTGGACC-3′, reverse, 5′-TCAGCCAGGTAAAYAAGGGC-3′. Housekeeping genes Pigo and Adck1 were quantified by PrimeTime quantitative PCR assays (Integrated DNA Technologies, Mm.PT.56a.41787370, Mm.PT.58.6147472). The hACE2 signal was normalized to Adck and Pigo.

### Ex vivo imaging

Increased glycolysis was assessed by imaging uptake of XenoLight RediJect 2-DG (RJ2-DG) into tissues in vivo. Two hours after i.v. injection was determined to be the optimal time point for imaging maximal signal and tissue distribution (data not shown), consistent with the manufacturer’s recommendations (PerkinElmer). RJ2-DG was injected i.v. (100 μl) via the retro-orbital sinus. After 2 h, mice were euthanized by isoflurane inhalation and whole organs were aseptically removed and placed in a 90-mm-diameter petri dish. Tissues were immediately imaged with an IVIS Lumina XR imaging system using the ICG (indocyanine green) filter. Images were analyzed using Living Image 4.0 (PerkinElmer). Regions of interest were drawn around the excised tissue, fluorescent signal intensity was measured, and total radiance efficiency was determined.

### Mass spectrometry

For all liquid chromatography–mass spectrometry (LC-MS) analyses, LC-MS–grade solvents and reagents were used. The postcaval lobes of the mice were collected into 400 μl of ice-cold methanol. Lung lobes were subsequently homogenized, and 400 μl of water was added. Alternatively, whole blood was isolated by terminal bleed and 100 μl was added directly to 100 μl of cold methanol. To the suspension, 400 μl of chloroform was added and the sample was agitated via shaking at 4°C for 20 min. Layering was induced by centrifugation at 16,000 × *g*, 4°C for 20 min. The polar (upper) layer was collected and diluted 1:10 in 50% methanol in water. Analysis was performed consistent with previous studies (3). Samples were injected onto a Sciex ExionLC AC system and separated using an ion pairing strategy on a Waters Atlantis T3 column (100 Å, 3 μm, 3 × 100 mm) with a 15-min gradient from 5 mM tributylamine, 5 mM acetic acid in 2% isopropanol, 5% methanol, 93% water (v/v) to 100% isopropanol. Metabolites were detected on a Sciex 5500 QTRAP mass spectrometer using a multiple reaction monitoring strategy consistent with previous reports (3). Quality control injections were performed after every 10 injections to ensure instrument stability. All data were processed and filtered using MultiQuant software 3.0.3 (Sciex). A 50% missing value cutoff and 30% quality control coefficient of variance cutoff were imposed. Remaining missing values were replaced with the minimum group value for that feature. Following filtering, the log_2_ fold change in metabolite levels was calculated relative to mock-infected + vehicle-treated controls. All univariate and multivariate analyses were performed in MarkerView software 1.3.1 (Sciex).

### Statistical analysis

Statistical significance between group means was determined where indicated using a Student *t* test or one- or two-way ANOVA followed by Sidak or Dunnett multiple comparison tests to compensate for type I error. An unpaired, nonparametric Mann–Whitney *U* test was used for comparisons of histopathological scores. A log-rank Mantel–Cox test was used to evaluate significance within survival studies between vehicle- and DMOG-treated groups. Statistical power was greater than <0.8 to determine sample size, and statistical significance was defined as a probability of type I error occurring at a *p* value of <0.05. All in vitro studies were repeated a minimum of three to four times (*n* = 3–4), and in vivo studies were repeated twice with four to six mice per group for each experimental replicate. Data were pooled (*n* = 8–11 mice total) for statistical analysis. For analysis of mass spectrometry, metabolites with divergent patterns were filtered using a Benjamini–Hochberg correction with application of a 20% and more stringent 10% false discovery rate. All statistical analysis was performed using GraphPad Prism 9.0.

## Results

### SARS-CoV-2 infection maintains mitochondrial function early following infection

**FIGURE 1.**
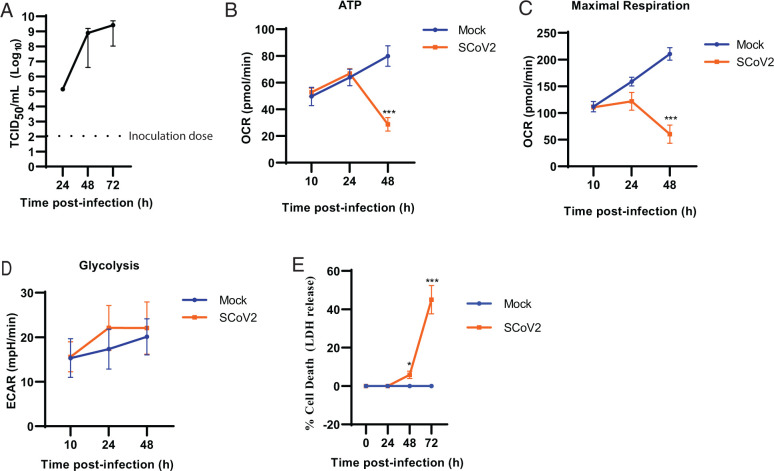
SARS-CoV-2 infection maintains mitochondrial function early following infection. (**A**) hACE2-A549 cells were infected with SARS-CoV-2 (MOI of 0.01) or mock (uninfected) and assessed for viral burden by TCID_50_. (**B** and **C**) Extracellular flux analysis of mitochondrial function was performed to determine the ATP-linked oxygen consumption rate (OCR) after injection of oligomycin (B) and maximal respiration was calculated after injection of FCCP (C). (**D**) Glycolysis was determined using the glycolysis stress test. (**E**) Cell death as determined by extracellular LDH activity over time following SARS-CoV-2 infection. Data are shown as mean ± SEM from four pooled experiments (*n* = 4) with six technical replicates per group. *p < 0.05, ***p < 0.001 indicate significance between mock and SARS-CoV-2 infection by a Student *t* test or one-way ANOVA followed by a Dunnett multiple comparison test. ECAR, extracellular acidification rate.

To model SARS-CoV-2 infection of lung epithelial cells in vitro, we used A549 cells engineered by lentiviral transfection to express hACE2, leading to increased susceptibility to infection (16). A549 cells are a human lung epithelial cell line that were specifically selected for these studies based on their known ability to undergo HIF-1α–dependent metabolic reprogramming (18). We characterized our in vitro model by examining viral burden, cellular fate, and bioenergetics over time. As expected, viral burden increased to 72 h postinfection (Fig. 1A). Mitochondrial flux, as measured by cellular OCR, was maintained in infected cells, including ATP-synthase limited respiration and maximal respiration rates during the first 24 h of infection (Fig. 1B, 1C). In contrast, at 48 h postinfection, mitochondrial ATP levels and maximal respiration rates rapidly decreased, indicating loss of bioenergetic activity (Fig. 1B, 1C). Surprisingly, SARS-CoV-2 infection did not trigger increased glycolysis, even as mitochondrial function started to decline in host cells (Fig. 1D). The absence of a transition to glycolysis suggested that A549 cells were not undergoing metabolic reprogramming consistent with dysregulation in the α-ketoglutarate metabolism and associated HIF-1α stabilization through PHD. Ultimately, infected cells entered a state of mitochondrial collapse between 48 and 72 h postinfection corresponding to increased cell death (Fig. 1E).

### SARS-CoV-2 infection limits HIF-1α stabilization and manipulation of cellular bioenergetics

**FIGURE 2.**
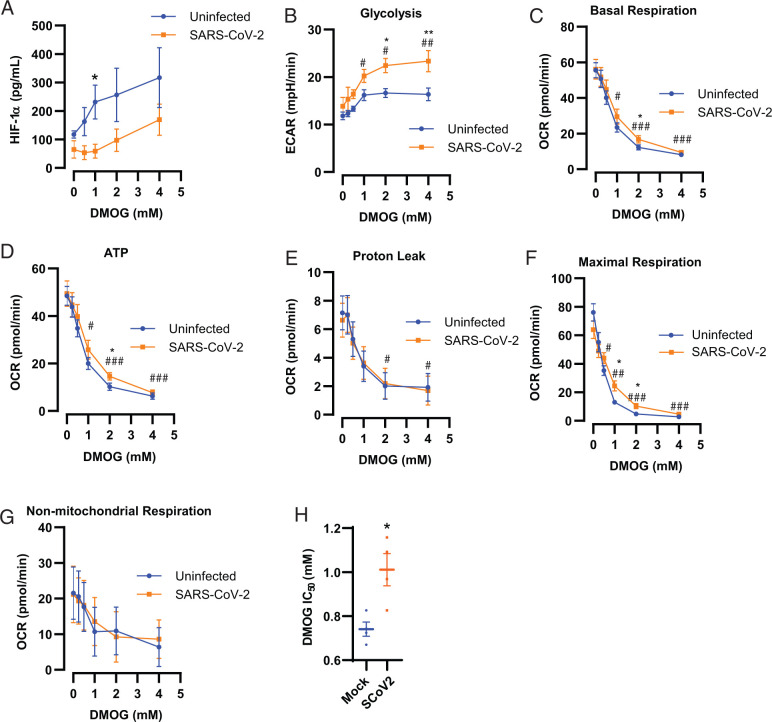
DMOG treatment overcomes SARS-CoV-2 manipulation of cellular bioenergetics. hACE2-A549 cells were infected with SARS-CoV-2 (MOI of 0.01) or uninfected (mock) and treated with increasing doses of DMOG. (**A**) Intracellular HIF-1α levels were measured by ELISA. (**B**–**G**) Mitochondrial or glycolytic stress tests via extracellular flux analysis were also performed at 24 h postinfection to determine (B) glycolytic extracellular acidification rate (ECAR), (C) basal respiration, (D) ATP-dependent oxygen consumption rate (OCR), (E) proton leak, (F) maximal respiration, and (G) nonmitochondrial respiration rates. DMOG (0 mM) represents the baseline effects of infection or mock infection on cells after 24 h. (**H**) IC_50_ of DMOG inhibition of OCR on mock- or SARS-CoV-2–infected cells. For calculation of the IC_50_, each individual data point represents an experimental repeat. Data are shown as mean ± SEM from four pooled experiments (*n* = 4) with six technical replicates per group. ^#^*p* < 0.05, ^##^*p* < 0.01, ^###^*p* < 0.001 indicate significance relative to the baseline (0 mM DMOG) using a one-way ANOVA followed by a Dunnett multiple comparison test. *p < 0.05, **p < 0.01 indicates significance between mock (uninfected) or SARS-CoV-2–infected groups using a Student *t* test.

It is plausible that SARS-CoV-2 evades triggering stabilization of HIF-1α and associated metabolic reprogramming postinfection to limit cellular defense mechanisms. Accordingly, therapeutic interventions that allow for stabilization of HIF-1α may override SARS-CoV-2–associated manipulation and improve disease outcomes. One mechanism to enhance stabilization of HIF-1α is via inhibition of PHD. Therefore, we examined the therapeutic efficacy of DMOG, an α-ketoglutarate derivative and well-established competitive PHD inhibitor (19). Consistent with PHD inhibition, DMOG treatment increased HIF-1α stabilization in hACE2-expressing A549 cells in a dose-dependent manner (Fig. 2A, Supplemental Fig. 1). Similarly, DMOG treatment of SARS-CoV-2–infected A549 cells increased stabilization of HIF-1α at higher doses, although not to the same level as DMOG treatment in uninfected cells (Fig. 2A, Supplemental Fig. 1). Furthermore, we demonstrated that ACE2 expression in these cells was uncoupled from HIF-1α–mediated regulation, as indicated by the lack of change in ACE2 protein levels after PHD inhibition and/or infection (Supplemental Fig. 1). This feature allowed for examination of HIF-1α–mediated effects on infection independent of its reported role on viral uptake (14, 15), specifically with respect to metabolic outcomes. Consistent with increased HIF-1α stabilization, DMOG treatment in both mock- and SARS-CoV-2–infected cells was associated with increased glycolysis at 24 h postinfection (Fig. 2B). The effect of DMOG on glycolysis was exacerbated in infected cells at doses >1 mM, indicating that SARS-CoV-2 was not only incapable of limiting DMOG-induced upregulation of glycolysis but synergized to cause more flux.

Interestingly, HIF-1α stabilization was reduced in SARS-CoV-2 cells treated with DMOG at 24 h postinfection at intermediate doses, indicating an infection-associated shift in the apparent IC_50_ of this agent (Fig. 2A, Supplemental Fig. 1C). To further explore this relationship, we measured parameters of mitochondrial respiration as extensions of the effects of infection and DMOG treatment on HIF-1α stabilization. DMOG treatment resulted in significant dose-dependent inhibition of basal respiration, proton leak, ATP-driven respiration, and maximal respiration rates in both infected and uninfected cells treated with DMOG (Fig. 2C–G). Consistent with decreased stabilization of HIF-1α (Fig. 2A), resistance to the effects of DMOG was evident in SARS-CoV-2–infected cells at intermediate doses in the basal respiration, ATP-driven oxidative phosphorylation, and maximal respiration rates (Fig. 2C, 2D, 2F). Infection with DMOG treatment did not significantly affect decreased proton leak or nonmitochondrial respiration rates relative to uninfected controls (Fig. 2E, 2G). To further quantify the resistance of mitochondrial reprogramming to DMOG treatment in infected cells, the IC_50_ for DMOG against basal respiration was calculated and determined to be significantly increased (Fig. 2G). Taken together, these data support the active manipulation of the HIF-1α/PHD–mediated metabolic outcomes during infection.

### DMOG treatment limits SARS-CoV-2–induced cell death independent of viral burden

**FIGURE 3.**
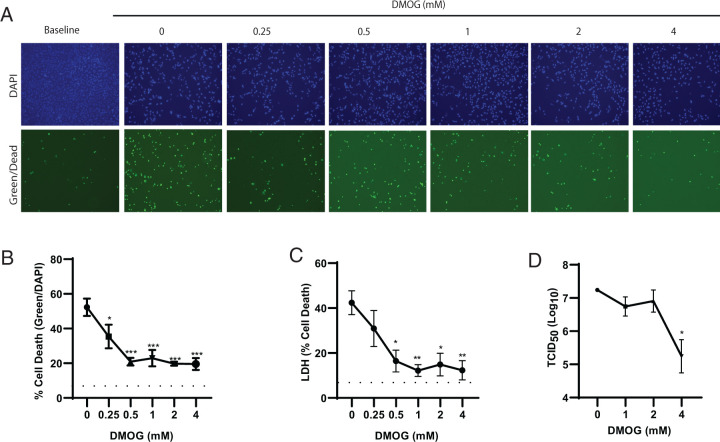
DMOG treatment limits SARS-CoV-2–induced cell death independent of viral burden. (**A**–**D**) hACE2-A549 cells were infected with SARS-CoV-2 (MOI of 0.01) and then treated with increasing doses of DMOG; cell death (A–C) or viral burden (D) was assessed after 72 h. The percent cell death by microscopic enumeration of NucGreen-positive cells was calculated. (A) Representative images of A549 cell cultures used to calculate (B). (C) Extracellular LDH activity as a surrogate marker of cell death. (D) Viral titer measure by TCID_50_. Data are shown as mean ± SEM from three pooled experiments (*n* = 3) with three technical replicates per group. The dotted lane represents the background cell death levels in uninfected cultures without DMOG treatment. *p < 0.05, **p < 0.01, ***p < 0.001 indicate significance relative to 0 (untreated) cells infected with SARS-CoV-2 using one-way ANOVA followed by a Dunnett multiple comparison test.

A primary function of HIF-1α stabilization is to avoid bioenergetic crisis that may lead to cell death (20). Therefore, the ability of DMOG to increase glycolysis and overcome SARS-CoV-2 metabolic manipulation or evasion strategies could translate into reduced cell death. We next assessed whether DMOG treatment reduced cell death in SARS-CoV-2–infected hACE2-A549 cells at 72 h postinfection. We observed a significant dose-dependent inhibition of cell death among infected cells treated with DMOG compared with vehicle-treated controls (Fig. 3A–C). Interestingly, control of cell death occurred independent of viral burden except for at the highest dose of DMOG assessed (Fig. 3D). Doses of DMOG >4 mM resulted in significant toxicity and reduced cellular replication (data not shown) and therefore were not further pursued. At the 4 mM dose, we observed less division of A549 cells, potentially leading to less target cells for SARS-CoV-2 to infect (data not shown), which could account for the reduced viral burden. Overall, these data support that enhanced survival of SARS-CoV-2–infected cells was due to direct effects on host cell metabolism and occur irrespective of viral burden.

### DMOG treatment improves survival in SARS-CoV-2–infected K18-hACE2 mice

**FIGURE 4.**
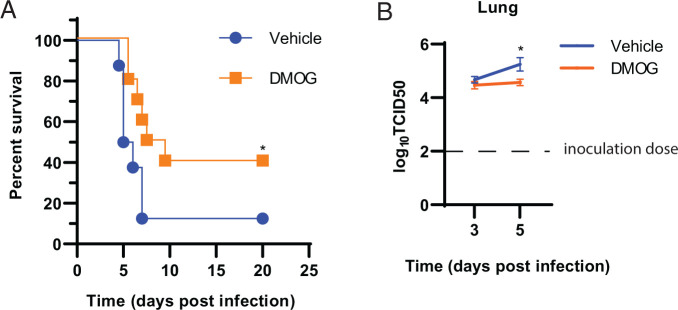
DMOG treatment improves survival in SARS-CoV-2–infected K18-hACE2 mice. (**A**) Survival of K18-hACE2 mice with either vehicle or DMOG treatment. (**B**) Viral loads in the lungs of infected mice on day 3 and 5 postinfection. Data are shown from two individual experiments pooled with a total of 8–11 mice per treatment group (*n* = 8–11). For (B), data are shown as mean ± SEM. *p < 0.05 indicates significance using a Mantel–Cox nonparametric test in (A). *p < 0.05 indicates significance by two-way ANOVA followed by a Sidak multiple comparison test in (B).

To address the in vivo efficacy of DMOG against SARS-CoV-2, we used the highly sensitive, lethal, K18-hACE2 mouse model (21). Treatment with DMOG resulted in a significant increase in survival, as well as an extension in mean time to death for mice that did not survive (mean of 6.92 d [DMOG] versus 5.64 d [vehicle]) compared with vehicle control (Fig. 4A). To elucidate potential mechanisms by which PHD inhibition improves outcomes postinfection, we first evaluated whether treatment had antiviral activity. DMOG treatment did not significantly change viral titers at early stages of infection (day 3) compared with vehicle controls. However, by day 5 postinfection we observed a partial but significant reduction in viral burden (Fig. 4B). HIF-1α stabilization has been implicated in the downregulation of ACE2 in the Syrian hamster model and in human cells as a primary mechanism responsible for reduced disease burden (14, 15). However, similar to the A549 cells used in this study, hACE2 expression in the K18-hACE2 mouse is not linked to its native promoter and is therefore uncoupled from canonical HIF-1α regulation (22). Therefore, it is unlikely that the modest effects observed on viral burden in this model would be a consequence of HIF-1α–mediated reduction in ACE2 levels. To confirm this, K18-hACE2 mice were treated with DMOG or vehicle during SARS-CoV-2 infection and lung tissues were assessed on day 5 postinfection for hACE2 levels by RT-PCR. As predicted, there was no significant negative impact of DMOG treatment on hACE2 transcription levels (Supplemental Fig. 2B). Taken together, and consistent with our in vitro findings, these data indicate that the protective effect of DMOG in SARS-CoV-2–infected mice was a result of targeting host responses rather than direct control of viral uptake.

### DMOG treatment increases glycolysis and limits metabolic programs triggered by SARS-CoV-2 in vivo

**FIGURE 5.**
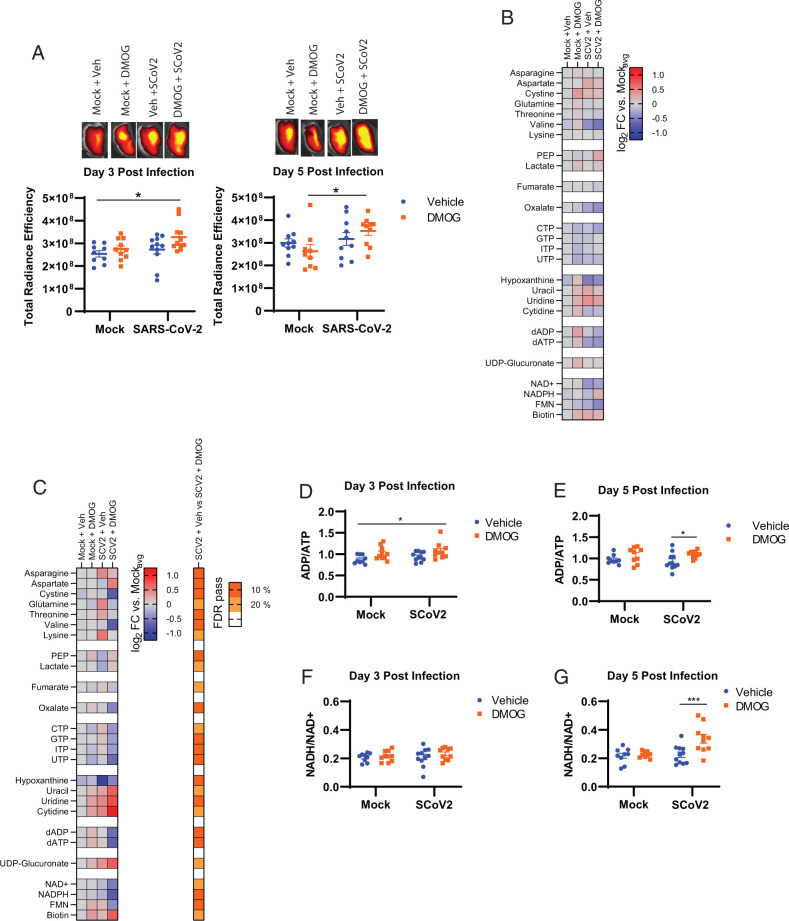
DMOG treatment increases glycolysis and limits metabolic programs triggered by SARS-CoV-2 in vivo. (**A**) Representative images and composite total radiance efficiency calculations for lungs of SARS-CoV-2–infected mice treated with vehicle or DMOG on day 3 and day 5 postinfection. (**B** and **C**) Targeted screen of soluble metabolite intermediates within major central metabolic pathways on day 3 (B) and day 5 (C) postinfection. (**D** and **E**) ADP/ATP ratios on day 3 (D) and day 5 (E) postinfection. (**F** and **G**) NADH/NAD^+^ on day 3 (F) and day 5 (G) postinfection. Data are shown as mean ± SEM from two individual experiments pooled with a total of 8–10 mice per treatment group (*n* = 8–11). For (A) and (D)–(G), *p < 0.05, ***p < 0.001 indicate significance by two-way ANOVA followed by a Sidak multiple comparison test. For (C), significance is indicated by Benjamini–Hochberg correction with false discovery rates of 10 and 20%.

Given the ability of DMOG to stabilize HIF-1α and increase glycolysis among SARS-CoV-2–infected cells in vitro, we hypothesized that the drug may be inducing a similar shift in metabolic responses in vivo. Therefore, we next assessed metabolic mechanisms by which DMOG may limit lethality following SARS-CoV-2 infection in vivo. To determine whether DMOG was shifting host cells toward a more glycolytic state in vivo, we measured uptake of the glucose derivative RJ2-DG in the lungs of infected and/or drug-treated mice as a surrogate marker for increased glycolysis (3). DMOG treatment in SARS-CoV-2–infected mice resulted in significantly increased RJ2-DG uptake compared with mock-infected and vehicle-treated controls on day 3 postinfection. Increased glycolysis in infected DMOG-treated animals was maintained on day 5 postinfection relative to DMOG treatment alone and trended upward relative to uninfected vehicle-treated controls (*p* = 0.08) (Fig. 5A). Consistent with increased glycolysis on day 5 postinfection, we observed increased levels of HIF-1α in the lungs of DMOG-treated mice infected with SARS-CoV-2 relative to SARS-CoV-2 infection alone (Supplemental Fig. 2C, 2D). Taken together, these data suggested that in agreement with our in vitro observations, DMOG treatment triggered early induction of metabolic reprogramming among SARS-CoV-2–infected mice and specifically increased glycolysis (Fig. 5A).

Measuring uptake of glucose can be a strong general indicator of a proinflammatory metabolic state (23). However, this technique does not capture broader alterations in central metabolism that may also be features of the protective efficacy of DMOG in vivo. To more deeply interrogate changes in host metabolic responses during SARS-CoV-2 infection in the presence and absence of DMOG, we next performed multitargeted metabolomics of central carbon metabolism in the lungs on day 3 and 5 postinfection using LC-MS as previously described (3). On day 3 postinfection, trends indicative of early upregulation of glycolysis including elevated phosphoenolpyruvate (PEP) and lactate levels were present but were not significant in DMOG-treated mice (Fig. 5B). However, on day 5 postinfection 26 metabolites out of 103 measured differed between vehicle and DMOG treatment in infected mice at the 20% false discovery rate threshold (Fig. 5B, 5C). In the absence of DMOG, SARS-CoV-2 infection resulted in reduced levels of glycolytic intermediates, accumulation of amino acids, and depletion of hypoxanthine (Fig. 5C), which was consistent with our previously reported findings among SARS-CoV-2–infected mice at later time points of infection (3). In contrast, DMOG treatment of SARS-CoV-2–infected mice reversed or limited infection-associated increases in asparagine, glutamine, threonine, and lysine, further depleted cystine and valine levels, and limited the loss of hypoxanthine (Fig. 5C). DMOG treatment also resulted in increased lactate, PEP, and ratios of ADP/ATP and NADH/NAD^+^ levels relative to vehicle-treated mice, further corroborating that PHD inhibition leads to metabolic changes associated with improved tissue bioenergetics (Fig. 5C–G). Lastly, we observed significant downregulation of purines and dinucleotide precursors, with concomitant increases in selected nucleobases (Fig. 5C). Taken together, these changes indicated that DMOG treatment resulted in selective regulation of amino acid metabolism and a shift in nucleotide salvage pathways (as indicated by decreased hypoxanthine). By extension, these observations may reflect alterations to metabolic networks critical for transcription and translation. Finally, these data show that PHD inhibition reversed metabolic manipulation or evasion programs associated with SARS-CoV-2 pathogenesis, correlating with increased survival, and reveal a role for modulation of host metabolic responses via destabilization of HIF-1α as an important feature of SARS-CoV-2 infection.

### DMOG treatment modifies the temporal dynamics of the host inflammatory response

**FIGURE 6.**
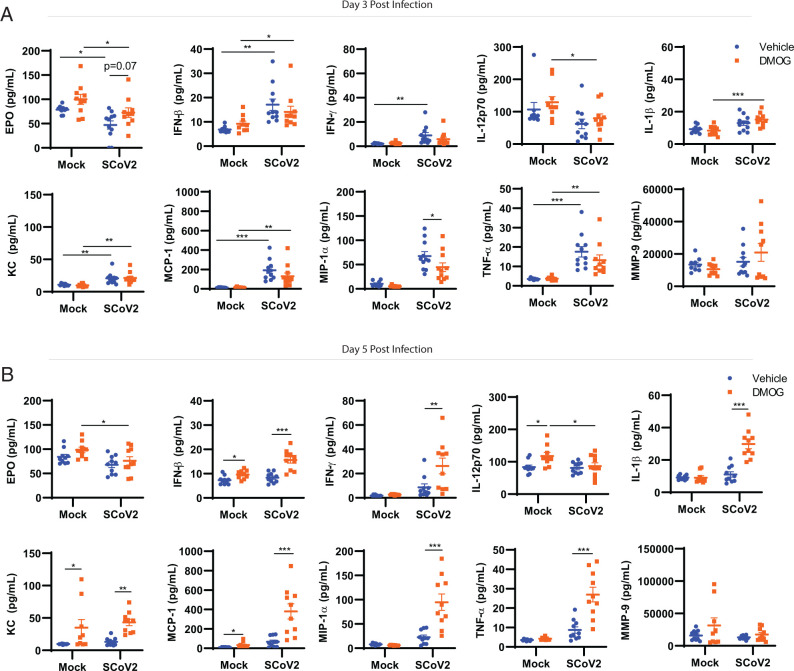
DMOG treatment modifies the temporal dynamics of the host inflammatory response. (**A** and **B**) Proinflammatory cytokines/chemokines, MMP-9, and EPO were measured on day 3 (A) and day 5 (B) postinfection. Data are shown as mean ± SEM from two individual experiments pooled with a total of 8–10 mice per treatment group (*n* = 8–11). *p < 0.05, **p < 0.01, ***p < 0.001 indicate significance by two-way ANOVA followed by a Sidak multiple comparison test.

As shown above, targeting PHD resulted in increased glycolysis among SARS-CoV-2–infected animals. In addition to this well-described metabolic adaption to oxygen limitation, PHD-dependent stabilization of HIF-1α may also initiate immune programs to respond to tissue damage (24). Therefore, in addition to overcoming metabolic defects triggered by SARS-CoV-2, DMOG treatment could also allow for activation of beneficial host innate immune function. To address this possibility, we measured an array of 10 proinflammatory and or immune modulatory effectors in the lungs of infected mice with or without DMOG treatment over time. Vehicle-treated, SARS-CoV-2–infected mice had a significant upregulation of inflammatory mediators on day 3 postinfection (Fig. 6A). At this time point, DMOG treatment selectively reduced MIP-1α levels and decreased, although not significantly, MCP-1, TNF-α, IFN-γ, and IFN-β levels among infected mice, indicating a modest early suppressive effect. We measured erythropoietin (EPO) postinfection and observed significantly lower levels by day 3 postinfection irrespective of DMOG treatment. However, although not statistically significant, treatment with DMOG increased basal EPO levels in the lung, which appeared to be sustained with infection (*p* = 0.07) (Fig. 6A). By day 5 postinfection the immune-suppressed phenotype was reversed and SARS-CoV-2–infected mice treated with DMOG had significant increases in all effectors measured except for EPO, MMP-9, and IL-12p70 (Fig. 6B). There was no significant effect of DMOG treatment on MMP-9 or IL-12p70 levels in the context of infection in the lungs (Supplemental Fig. 2C, 2D). Consistent with an increased broader systemic inflammatory response, SARS-CoV-2 infection with DMOG treatment resulted in significant exacerbation of specific circulating mediators, including IL-12p70, TNF-α, and MCP-1, in the serum on day 5 postinfection relative to vehicle-treated mice (Supplemental Fig. 3). Interestingly, DMOG treatment reduced serum EPO levels in SARS-CoV-2–infected mice relative to vehicle treatment (Supplemental Fig. 3). In addition to molecular markers of inflammation, we determined whether DMOG treatment, as well as the resultant enhanced inflammatory profile present in the lungs, impacted SARS-CoV-2–induced pathology by histological analysis. No significant changes in pathology among vehicle- or DMOG-treated SARS-CoV-2–infected animals was observed (Supplemental Fig. 4). Therefore, the enhanced inflammatory profile generated in SARS-CoV-2–infected mice with DMOG treatment did not result in exacerbated or other clear changes in pathology.

## Discussion

In the current study we provide evidence that SARS-CoV-2 infection limits the stabilization of HIF-1α and associated downstream effects on metabolic reprograming through α-ketoglutarate sensing 2-OGDD. We then demonstrated in a preclinical model that augmenting the HIF-1α circuit via 2-OGDD inhibition with DMOG improved survival in the highly sensitive K18-hACE2 mouse model of SARS-CoV-2. Furthermore, we provided mechanistic support for how DMOG treatment acts to promote metabolic reprogramming in the host to overcome manipulation and evasion strategies used by the virus in the absence of alteration of expression of a key receptor required for viral entry. In addition to survival, chemically induced enhancement of metabolic reprogramming in the host correlated with greater proinflammatory immune activity and partial reduction in viral burden at later stages of disease.

Targeting PHD/HIF-1α signaling to treat SARS-CoV-2 has received serious consideration based on improved outcomes in noninfection models of lung injury (6, 25–28). Justification for use of PHD inhibitors to treat infection has been largely based on their ability to improve insufficient or dysregulated HIF-1α–mediated metabolic adaptations to hypoxia and anemia (6). The PHD inhibitor vadadustat, which is an α-ketoglutarate mimetic akin to DMOG, was developed to treat anemia and conditions with HIF-1α dysregulation (29). Early during the pandemic, a phase II clinical trial focused on treating hospitalized COVID-19 patients diagnosed with hypoxia with vadadustat was established and is currently ongoing (30). Findings from the current study may inform ongoing clinical trials and help refine therapeutic strategies to improve outcomes. For example, it is important that HIF-1α–associated metabolic adaptation to infection includes upregulation of glycolysis to maintain intracellular ATP and NADH needed for tissue function and host immune activation (31). Interestingly, this specific metabolic adaption was not elicited at early time points after SARS-CoV-2 infection as shown in the current study and in prior reports (3). Furthermore, resistance to HIF-1α stabilization was evident by a significant increase in the IC_50_ associated with DMOG inhibition of oxidative phosphorylation in the presence of infection, suggesting that this critical pathway for detection of metabolic stress was limited by SARS-CoV-2. Treatment with DMOG was sufficient to overcome SARS-CoV-2 evasion of central metabolic programs including glycolysis, which may account for its cellular protective activity through maintenance of bioenergetic intermediates. Considering our findings showing limited HIF-1α activation with SARS-CoV-2 infection, as well as reports that lower HIF-1α levels in tissues correlated with increased tissue damage (13), timely application of α-ketoglutarate–derived inhibitors to drive HIF-1α–associated metabolic adaptations may be needed to effectively promote tissue-protective responses.

An unexpected consequence of DMOG treatment was the enhancement of host inflammatory cytokines (Fig. 6). Enhanced inflammation has been considered a potential risk of 2-OGDD inhibitors and their application in COVID-19, as several studies have shown that inflammation is positively correlated with worse outcomes (6). To address this concern, we assessed tissue pathology by histology and did not observe any adverse outcomes associated with DMOG treatment despite enhanced cytokine/chemokine levels (Supplemental Fig. 4). Therefore, timing, specific localization, and overall composition of the inflammatory milieu may be a more important determinant of severe disease rather than increased inflammation in the broader context in the K18-hACE2 mouse model. The notion of increased inflammation driving worse outcomes has further been challenged in recent reports comparing COVID-19 to influenza-infected populations, among other studies (32–35). Consistent with this conclusion, we have previously demonstrated that most proinflammatory cytokines/chemokines are reduced at later stages of SARS-CoV-2 infection in mice and therefore are not reliably predictive of the onset of morbidity (3). Rather, metabolic dysregulation and specific tissue-damaging inflammation associated with the pulmonary vasculature were better indicators of morbidity (3). Therefore, timely DMOG-induced augmentation of metabolic reprogramming and the specific inflammatory milieu in infected mice may promote a more effective host response that limits pathophysiological outcomes that contribute to morbidity.

Recent studies have implicated HIF stabilization following PHD inhibition or that hypoxia promotes antiviral activity as a primary mechanism against SARS-CoV-2. Specifically, hypoxia or treatment with the PHD inhibitor roxadustat are reported to limited ACE2 and TMPRSS2 expression and SARS-CoV-2 RNA transcription in primary epithelial cells or hamster models, resulting in dampened viral entry and replication (14, 15). In contrast to these prior reports, we did not observe direct antiviral activity after DMOG treatment in models used in the current study. This was a consequence of the uncoupling of human ACE2 expression from HIF-1α regulation in vitro (A549 cells) and in vivo (K18-hACE2 mice). With the potential contribution of ACE2 downregulation by HIF-1α bypassed, the therapeutic effects of DMOG more directly toward host metabolism could be interrogated. Under these conditions, DMOG treatment was revealed to promote prosurvival metabolic and inflammatory responses. Furthermore, differences in bioavailability or specificity between DMOG versus other HIF modulators, such as roxadustat, exist that could contribute to reported outcomes (36). DMOG is structurally the most basic antagonist developed among available PHD inhibitors, with the greatest similarity to α-ketoglutarate. Furthermore, DMOG is reported to act on mitochondrial function prior to its activity against HIF signaling (37). Additionally, DMOG is also reported to act on epigenetic regulatory enzymes that sense α-ketoglutarate or are sensitive to acetyl-CoA levels, as well as autophagy (8, 9, 38). Alternatively, roxadustat is reported to have higher bioavailability in target tissues with more specificity to HIF-1 signaling (39). Therefore, in addition to HIF-dependent effects, DMOG may also promote HIF-independent effects on mitochondrial metabolism, epigenetic enzymes, or autophagy through regulation of α-ketoglutarate that may also contribute to its therapeutic efficacy in lethal SARS-CoV-2 infection models.

An important aspect of our study was the interrogation of how DMOG treatment influenced changes in the metabolic milieu in the lung. Changes in specific pools of metabolites can indicate aberrant or protective host responses to infection. For example, we have previously reported that increased amino acid accumulation in the lungs of SARS-CoV-2–infected mice was associated with onset of morbidity (3). These findings were recapitulated on day 5 postinfection in the current study (Fig. 5C). Specific reasons for accumulation of selected amino acids in the lungs after SARS-CoV-2 infection are poorly understood but may reflect an increase in nutrient supply needed to maintain viral replication. Asparagine availability has been specifically implicated as a limiting factor in vaccinia virus and CMV replication (40). We observed specific reversal of levels of metabolites associated with asparagine de novo biosynthesis, including aspartate and glutamate, with DMOG treatment, suggesting that PHD inhibition may limit the supply of important viral building blocks (Fig. 5C). PHD also acts as an important sensor of amino acid metabolism through detection of its substrate α-ketoglutarate (41). The tricarboxylic acid cycle through glutaminolysis and histidine, arginine, or proline catabolism provide important sources of α-ketoglutarate sensed by PHD. A potential consequence of accumulation of amino acids in SARS-CoV-2 infection may be a concomitant increase in enzymatic activity of PHD through increased α-ketoglutarate levels, thereby limiting HIF-1α stabilization. This mechanism may also account for the significant shift in the IC_50_ needed for DMOG to induce metabolic reprogramming observed in A549 cells in vitro (Fig. 2F).

In addition to the effect DMOG had on the amino acids described above, the ability of DMOG treatment to reverse the loss of hypoxanthine in the lungs after SARS-CoV-2 infection is a promising signal of its therapeutic potential. Increased hypoxanthine levels in the serum have been reported as a biomarker of disease severity with acute lung injury and ARDS (42). Measurements by our group and others have demonstrated that serum levels of hypoxanthine and its downstream metabolites xanthine and urate were all correlates of severe COVID-19 and poor patient outcome (3, 43–45). Despite this strong correlation, little is understood regarding how hypoxanthine levels change in the lungs after viral infection. Hypoxanthine plays a critical role in primarily two metabolic networks. First, hypoxanthine-guanine phosphoribosyltransferase can convert hypoxanthine to inosine monophosphate as part the nucleotide salvage pathway (46). Alternatively, xanthine oxidase can convert hypoxanthine to uric acid and in the processes generate ROS (36). Decreased availability of hypoxanthine after SARS-CoV-2 infection may restrict activity of one or both pathways in the lungs of infected mice, leading to limited repair of DNA damage and ROS production. Treatment with DMOG could re-establish these pathways, leading to increased ROS and/or DNA repair, thereby limiting purine bioavailability to the virus. HIF-1α stabilization and regulation of purine nucleoside salvage has been reported to protect neurons from hypoxic injury (47), potentially indicating that a similar response may be at work in pulmonary or vasculature tissues.

In addition to the positive effect that 2-OGDD inhibition had on modulating central carbon metabolism, treatment with DMOG may provide the added benefit of driving physiological adaptations to counter hypoxic anemia (6). Increased HIF-1α stabilization is reported to promote increased EPO levels, leading to RBC production, changes in vascular tone, increased oxygen carrying capacity, and other angiogenic activities (48, 49). Correlative support for this hypothesis is found in populations that reside at high elevations, which typically retain higher baseline HIF-1α activity and EPO levels and had less severe SARS-CoV-2 outcomes (50). Conversely, decreased EPO levels in populations at lower elevations were associated with worse outcomes in SARS-CoV-2 (48, 49). Furthermore, i.v. delivery of EPO has been reported to improve outcomes in a COVID-19 case report (51). The effects of DMOG on EPO levels did not achieve statistical significance in the lungs in this study; however, we did observe a strong trend in which EPO levels were increased with DMOG treatment on day 3 postinfection (Supplemental Fig. 2A). However, the reverse outcome was evident in the serum, with DMOG treatment significantly reducing serum EPO levels in SARS-CoV-2–infected mice relative to vehicle treatment by day 5 postinfection (Supplemental Fig. 3). Therefore, the specific contribution of EPO to survival in the K18-hACE2 mouse remains to be elucidated.

Despite our promising findings for impairing 2-OGDD as an effective strategy for treatment of SARS-CoV-2, there have been several speculated negative effects of 2-OGDD inhibition (6). In human monocytes, SARS-CoV-2 replication is reported to increase glycolysis through a HIF-1α–dependent mechanism in ex vivo cultures, leading to enhanced viral replication, increased epithelial cell death, and reduced T cell function (52). Monocytes are not considered a primary target cell of SARS-CoV-2 during early stages of disease, but they may contribute to disease severity at later stages (53). One limitation of the current work is that impacts of DMOG treatment were interrogated at the broader tissue level and we did not examine effects on specific cellular subsets, such as monocytes. However, our overall findings in the lungs support a protective role for DMOG with no evidence of enhancement of viral replication or tissue damage. Therefore, if enhanced viral replication specifically occurred in monocytes, it had negligible impact on overall disease severity in the K18-hACE2 mouse model. Alternatively, the potential for HIF-1α to increase urokinase plasminogen activator (uPA)/uPA receptor system signaling, which has been correlated with poor outcomes in SARS-CoV-2 patients, has been suggested (54). Although we did not measure uPA/uPA receptor activation, we did not observe any pathological features in the lungs or vasculature consistent with adverse outcomes, including enhanced tissue damage, in DMOG-treated mice. However, we did not examine extrapulmonary tissues that may be impacted, including the cardiovascular system or kidney. Ultimately, more studies are needed examining potential adverse outcomes associated with 2-OGDD inhibition in vivo and in ongoing clinical trials.

In conclusion, this study identifies an immunometabolic suppression mechanism in SARS-CoV-2 infection that blocks appropriate maturation of the HIF-1α response through α-ketoglutarate sensing by 2-OGDDs. We provide preclinical proof-of-principle and mechanistic support for 2-OGDD inhibition as a viable therapeutic strategy against SARS-CoV-2 infection that involves augmentation of the HIF-1α response independent of regulation of receptors required for viral entry in target host cells. We also characterize specific metabolic and inflammatory effects of DMOG in the presence of SARS-CoV-2 infection and show that certain infection-induced patterns are overcome, allowing for a superior metabolic and immune response to the virus. Ultimately, these results advance our understanding of the metabolic regulation of disease pathogenesis after SARS-CoV-2 infection and open the window to development of additional metabolism-based therapeutic intervention strategies.

## Supplementary Material

Supplemental Figures 1 (PDF)

## References

[1] Boutou, A. K., A. Asimakos, E. Kortianou, I. Vogiatzis, A. Tzouvelekis. 2021. Long COVID-19 pulmonary sequelae and management considerations. J. Pers. Med. 11: 838. 34575615 10.3390/jpm11090838 PMC8469288

[2] Russell, C. D., N. I. Lone, J. K. Baillie. 2023. Comorbidities, multimorbidity and COVID-19. Nat. Med. 29: 334–343. 36797482 10.1038/s41591-022-02156-9

[3] Jessop, F., B. Schwarz, D. Scott, L. M. Roberts, E. Bohrnsen, J. R. Hoidal, C. M. Bosio. 2022. Impairing RAGE signaling promotes survival and limits disease pathogenesis following SARS-CoV-2 infection in mice. JCI Insight 7: e155896. 35076028 10.1172/jci.insight.155896 PMC8855831

[4] Cevik, M., K. Kuppalli, J. Kindrachuk, M. Peiris. 2020. Virology, transmission, and pathogenesis of SARS-CoV-2. BMJ 371: m3862. 33097561 10.1136/bmj.m3862

[5] Jia, H., C. Liu, D. Li, Q. Huang, D. Liu, Y. Zhang, C. Ye, D. Zhou, Y. Wang, Y. Tan, . 2022. Metabolomic analyses reveal new stage-specific features of COVID-19. Eur. Respir. J. 59: 2100284. 34289974 10.1183/13993003.00284-2021 PMC8311281

[6] Poloznikov, A. A., S. A. Nersisyan, D. M. Hushpulian, E. H. Kazakov, A. G. Tonevitsky, S. V. Kazakov, V. I. Vechorko, S. V. Nikulin, J. A. Makarova, I. G. Gazaryan. 2021. HIF prolyl hydroxylase inhibitors for COVID-19 treatment: pros and cons. Front. Pharmacol. 11: 621054. 33584306 10.3389/fphar.2020.621054 PMC7878396

[7] Ajaz, S., M. J. McPhail, K. K. Singh, S. Mujib, F. M. Trovato, S. Napoli, K. Agarwal. 2021. Mitochondrial metabolic manipulation by SARS-CoV-2 in peripheral blood mononuclear cells of patients with COVID-19. Am. J. Physiol. Cell Physiol. 320: C57–C65. 33151090 10.1152/ajpcell.00426.2020 PMC7816428

[8] Fong, G. H., K. Takeda. 2008. Role and regulation of prolyl hydroxylase domain proteins. Cell Death Differ. 15: 635–641. 18259202 10.1038/cdd.2008.10

[9] Islam, M. S., T. M. Leissing, R. Chowdhury, R. J. Hopkinson, C. J. Schofield. 2018. 2-Oxoglutarate-dependent oxygenases. Annu. Rev. Biochem. 87: 585–620. 29494239 10.1146/annurev-biochem-061516-044724

[10] Lee, S. H., M. Golinska, J. R. Griffiths. 2021. HIF-1-independent mechanisms regulating metabolic adaptation in hypoxic cancer cells. Cells 10: 2371. 34572020 10.3390/cells10092371 PMC8472468

[11] Weidemann, A., R. S. Johnson. 2008. Biology of HIF-1α. Cell Death Differ. 15: 621–627. 18259201 10.1038/cdd.2008.12

[12] Seki, S. M., A. Gaultier. 2017. Exploring non-metabolic functions of glycolytic enzymes in immunity. Front. Immunol. 8: 1549. 29213268 10.3389/fimmu.2017.01549 PMC5702622

[13] Wang, B. J., S. Vadakke-Madathil, L. B. Croft, R. I. Brody, H. W. Chaudhry. 2022. HIF1α cardioprotection in COVID-19 patients. JACC Basic Transl. Sci. 7: 67–69. 35097242 10.1016/j.jacbts.2021.12.001 PMC8785957

[14] Wing, P. A. C., T. P. Keeley, X. Zhuang, J. Y. Lee, M. Prange-Barczynska, S. Tsukuda, S. B. Morgan, A. C. Harding, I. L. A. Argles, S. Kurlekar, . 2021. Hypoxic and pharmacological activation of HIF inhibits SARS-CoV-2 infection of lung epithelial cells. Cell Rep. 35: 109020. 33852916 10.1016/j.celrep.2021.109020 PMC8020087

[15] Wing, P. A. C., M. Prange-Barczynska, A. Cross, S. Crotta, C. Orbegozo Rubio, X. Cheng, J. M. Harris, X. Zhuang, R. L. Johnson, K. A. Ryan, . 2022. Hypoxia inducible factors regulate infectious SARS-CoV-2, epithelial damage and respiratory symptoms in a hamster COVID-19 model. PLoS Pathog. 18: e1010807. 36067210 10.1371/journal.ppat.1010807 PMC9481176

[16] Scroggs, S. L. P., D. K. Offerdahl, D. P. Flather, C. N. Morris, B. L. Kendall, R. M. Broeckel, P. A. Beare, M. E. Bloom. 2020. Fluoroquinolone antibiotics exhibit low antiviral activity against SARS-CoV-2 and MERS-CoV. Viruses 13: 8. 33374514 10.3390/v13010008 PMC7822115

[17] Jessop, F., B. Schwarz, E. Heitmann, R. Buntyn, T. Wehrly, C. M. Bosio. 2018. Temporal manipulation of mitochondrial function by virulent *Francisella tularensis* to limit inflammation and control cell death. Infect. Immun. 86: e00044-18. 29760217 10.1128/IAI.00044-18 PMC6056872

[18] Luo, F., X. Liu, N. Yan, S. Li, G. Cao, Q. Cheng, Q. Xia, H. Wang. 2006. Hypoxia-inducible transcription factor-1α promotes hypoxia-induced A549 apoptosis via a mechanism that involves the glycolysis pathway. BMC Cancer 6: 26. 16438736 10.1186/1471-2407-6-26 PMC1402310

[19] Bruick, R. K., S. L. McKnight. 2001. A conserved family of prolyl-4-hydroxylases that modify HIF. Science 294: 1337–1340. 11598268 10.1126/science.1066373

[20] Greijer, A. E., E. van der Wall. 2004. The role of hypoxia inducible factor 1 (HIF-1) in hypoxia induced apoptosis. J. Clin. Pathol. 57: 1009–1014. 15452150 10.1136/jcp.2003.015032 PMC1770458

[21] McCray, P. B., Jr., L. Pewe, C. Wohlford-Lenane, M. Hickey, L. Manzel, L. Shi, J. Netland, H. P. Jia, C. Halabi, C. D. Sigmund, . 2007. Lethal infection of K18-hACE2 mice infected with severe acute respiratory syndrome coronavirus. J. Virol. 81: 813–821. 17079315 10.1128/JVI.02012-06 PMC1797474

[22] Winkler, E. S., R. E. Chen, F. Alam, S. Yildiz, J. B. Case, M. B. Uccellini, M. J. Holtzman, A. Garcia-Sastre, M. Schotsaert, M. S. Diamond. 2022. SARS-CoV-2 causes lung infection without severe disease in human ACE2 knock-in mice. J. Virol. 96: e0151121. 34668780 10.1128/JVI.01511-21 PMC8754206

[23] de Prost, N., M. R. Tucci, M. F. Melo. 2010. Assessment of lung inflammation with 18F-FDG PET during acute lung injury. AJR Am. J. Roentgenol. 195: 292–300. 20651183 10.2214/AJR.10.4499 PMC3172046

[24] Chen, Y., T. Gaber. 2021. Hypoxia/HIF modulates immune responses. Biomedicines 9: 260. 33808042 10.3390/biomedicines9030260 PMC8000289

[25] Tojo, K., N. Tamada, Y. Nagamine, T. Yazawa, S. Ota, T. Goto. 2018. Enhancement of glycolysis by inhibition of oxygen-sensing prolyl hydroxylases protects alveolar epithelial cells from acute lung injury. FASEB J. 32: 2258–2268. 32172532 10.1096/fj.201700888R

[26] Eckle, T., K. Brodsky, M. Bonney, T. Packard, J. Han, C. H. Borchers, T. J. Mariani, D. J. Kominsky, M. Mittelbronn, H. K. Eltzschig. 2013. HIF1A reduces acute lung injury by optimizing carbohydrate metabolism in the alveolar epithelium. PLoS Biol. 11: e1001665. 24086109 10.1371/journal.pbio.1001665 PMC3782424

[27] Ahn, J. M., S. J. You, Y. M. Lee, S. W. Oh, S. Y. Ahn, S. Kim, H. J. Chin, D. W. Chae, K. Y. Na. 2012. Hypoxia-inducible factor activation protects the kidney from gentamicin-induced acute injury. PLoS One 7: e48952. 23145036 10.1371/journal.pone.0048952 PMC3493596

[28] Nagamine, Y., K. Tojo, T. Yazawa, S. Takaki, Y. Baba, T. Goto, K. Kurahashi. 2016. Inhibition of prolyl hydroxylase attenuates Fas ligand-induced apoptosis and lung injury in mice. Am. J. Respir. Cell Mol. Biol. 55: 878–888. 27494234 10.1165/rcmb.2015-0266OC

[29] Chertow, G. M., P. E. Pergola, Y. M. K. Farag, R. Agarwal, S. Arnold, G. Bako, G. A. Block, S. Burke, F. P. Castillo, A. G. Jardine, PRO2TECT Study Group. 2021. Vadadustat in patients with anemia and non-dialysis-dependent CKD. N. Engl. J. Med. 384: 1589–1600. 33913637 10.1056/NEJMoa2035938

[30] Bobrow, B. J. 2020. Vadadustat for the prevention and treatment of acute respiratory distress syndrome (ARDS) in hospitalized patients with coronavirus disease 2019 (COVID-19). In: ClinicalTrials.gov. National Library of Medicine (US), Bethesda, MD. NLM Identifier: NCT04478071. Available at: https://clinicaltrials.gov/show/NCT04478071. Accessed: February 2, 2023.

[31] Kierans, S. J., C. T. Taylor. 2021. Regulation of glycolysis by the hypoxia-inducible factor (HIF): implications for cellular physiology. J. Physiol. 599: 23–37. 33006160 10.1113/JP280572

[32] Schwarz, B., L. M. Roberts, E. Bohrnsen, F. Jessop, T. D. Wehrly, C. Shaia, C. M. Bosio. 2022. Contribution of lipid mediators in divergent outcomes following acute bacterial and viral lung infections in the obese host. J. Immunol. 209: 1323–1334. 36002235 10.4049/jimmunol.2200162 PMC9529825

[33] Mudd, P. A., J. C. Crawford, J. S. Turner, A. Souquette, D. Reynolds, D. Bender, J. P. Bosanquet, N. J. Anand, D. A. Striker, R. S. Martin, . 2020. Distinct inflammatory profiles distinguish COVID-19 from influenza with limited contributions from cytokine storm. Sci. Adv. 6: eabe3024. 33187979 10.1126/sciadv.abe3024 PMC7725462

[34] Sinha, P., M. A. Matthay, C. S. Calfee. 2020. Is a “cytokine storm” relevant to COVID-19? JAMA Intern. Med. 180: 1152–1154. 32602883 10.1001/jamainternmed.2020.3313

[35] Weatherhead, J. E., E. Clark, T. P. Vogel, R. L. Atmar, P. A. Kulkarni. 2020. Inflammatory syndromes associated with SARS-CoV-2 infection: dysregulation of the immune response across the age spectrum. J. Clin. Invest. 130: 6194–6197. 33108354 10.1172/JCI145301 PMC7685746

[36] Hoidal, J. R., P. Xu, T. Huecksteadt, K. A. Sanders, K. Pfeffer, A. B. Sturrock. 1998. Lung injury and oxidoreductases. Environ. Health Perspect. 106(Suppl. 5): 1235–1239. 9788904 10.1289/ehp.98106s51235 PMC1533359

[37] Zhdanov, A. V., I. A. Okkelman, F. W. J. Collins, S. Melgar, D. B. Papkovsky. 2015. A novel effect of DMOG on cell metabolism: direct inhibition of mitochondrial function precedes HIF target gene expression. Biochim. Biophys. Acta 1847: 1254–1266. 26143176 10.1016/j.bbabio.2015.06.016

[38] Zhang, H., M. Bosch-Marce, L. A. Shimoda, Y. S. Tan, J. H. Baek, J. B. Wesley, F. J. Gonzalez, G. L. Semenza. 2008. Mitochondrial autophagy is an HIF-1-dependent adaptive metabolic response to hypoxia. J. Biol. Chem. 283: 10892–10903. 18281291 10.1074/jbc.M800102200 PMC2447655

[39] Hoppe, G., S. Yoon, B. Gopalan, A. R. Savage, R. Brown, K. Case, A. Vasanji, E. R. Chan, R. B. Silver, J. E. Sears. 2016. Comparative systems pharmacology of HIF stabilization in the prevention of retinopathy of prematurity. Proc. Natl. Acad. Sci. USA 113: E2516–E2525. 27091985 10.1073/pnas.1523005113 PMC4983815

[40] Pant, A., Z. Yang. 2020. Asparagine: an Achilles heel of virus replication? ACS Infect. Dis. 6: 2301–2303. 32786295 10.1021/acsinfecdis.0c00504

[41] Durán, R. V., E. D. MacKenzie, H. Boulahbel, C. Frezza, L. Heiserich, S. Tardito, O. Bussolati, S. Rocha, M. N. Hall, E. Gottlieb. 2013. HIF-independent role of prolyl hydroxylases in the cellular response to amino acids. Oncogene 32: 4549–4556. 23085753 10.1038/onc.2012.465 PMC3787797

[42] Quinlan, G. J., N. J. Lamb, R. Tilley, T. W. Evans, J. M. Gutteridge. 1997. Plasma hypoxanthine levels in ARDS: implications for oxidative stress, morbidity, and mortality. Am. J. Respir. Crit. Care Med. 155: 479–484. 9032182 10.1164/ajrccm.155.2.9032182

[43] Schwarz, B., L. Sharma, L. Roberts, X. Peng, S. Bermejo, I. Leighton, A. Casanovas-Massana, M. Minasyan, S. Farhadian, A. I. Ko, Yale IMPACT Team. 2021. Cutting edge: severe SARS-CoV-2 infection in humans is defined by a shift in the serum lipidome, resulting in dysregulation of eicosanoid immune mediators. J. Immunol. 206: 329–334. 33277388 10.4049/jimmunol.2001025 PMC7962598

[44] Doğan, H. O., O. Şenol, S. Bolat, Ş. N. Yıldız, S. A. Büyüktuna, R. Sarıismailoğlu, K. Doğan, M. Hasbek, S. N. Hekim. 2021. Understanding the pathophysiological changes via untargeted metabolomics in COVID-19 patients. J. Med. Virol. 93: 2340–2349. 33300133 10.1002/jmv.26716

[45] Valdés, A., L. O. Moreno, S. R. Rello, A. Orduña, D. Bernardo, A. Cifuentes. 2022. Metabolomics study of COVID-19 patients in four different clinical stages. Sci. Rep. 12: 1650. 35102215 10.1038/s41598-022-05667-0 PMC8803913

[46] Nyhan, W. L. 2005. Disorders of purine and pyrimidine metabolism. Mol. Genet. Metab. 86: 25–33. 16176880 10.1016/j.ymgme.2005.07.027

[47] zur Nedden, S., B. Tomaselli, G. Baier-Bitterlich. 2008. HIF-1 alpha is an essential effector for purine nucleoside-mediated neuroprotection against hypoxia in PC12 cells and primary cerebellar granule neurons. J. Neurochem. 105: 1901–1914. 18248612 10.1111/j.1471-4159.2008.05275.x PMC2992945

[48] Ehrenreich, H., K. Weissenborn, M. Begemann, M. Busch, E. Vieta, K. W. Miskowiak. 2020. Erythropoietin as candidate for supportive treatment of severe COVID-19. Mol. Med. 26: 58. 32546125 10.1186/s10020-020-00186-y PMC7297268

[49] Viruez-Soto, A., M. M. López-Dávalos, G. Rada-Barrera, A. Merino-Luna, D. Molano-Franco, A. Tinoco-Solorozano, N. Zubieta-DeUrioste, G. Zubieta-Calleja, C. Arias-Reyes, J. Soliz. 2021. Low serum erythropoietin levels are associated with fatal COVID-19 cases at 4,150 meters above sea level. Respir. Physiol. Neurobiol. 292: 103709. 34087493 10.1016/j.resp.2021.103709 PMC8169280

[50] Arias-Reyes, C., N. Zubieta-DeUrioste, L. Poma-Machicao, F. Aliaga-Raduan, F. Carvajal-Rodriguez, M. Dutschmann, E. M. Schneider-Gasser, G. Zubieta-Calleja, J. Soliz. 2020. Does the pathogenesis of SARS-CoV-2 virus decrease at high-altitude? Respir. Physiol. Neurobiol. 277: 103443. 32333993 10.1016/j.resp.2020.103443 PMC7175867

[51] Hadadi, A., M. Mortezazadeh, K. Kolahdouzan, G. Alavian. 2020. Does recombinant human erythropoietin administration in critically ill COVID-19 patients have miraculous therapeutic effects? J. Med. Virol. 92: 915–918. 32270515 10.1002/jmv.25839 PMC7262240

[52] Codo, A. C., G. G. Davanzo, L. B. Monteiro, G. F. de Souza, S. P. Muraro, J. V. Virgilio-da-Silva, J. S. Prodonoff, V. C. Carregari, C. A. O. de Biagi Junior, F. Crunfli, . 2020. Elevated glucose levels favor SARS-CoV-2 infection and monocyte response through a HIF-1α/glycolysis-dependent axis. [Published erratum appears in 2020 *Cell Metab.* 32: 498–499.] Cell Metab. 32: 437–446.e5. 32697943 10.1016/j.cmet.2020.07.007 PMC7367032

[53] Channappanavar, R., A. R. Fehr, R. Vijay, M. Mack, J. Zhao, D. K. Meyerholz, S. Perlman. 2016. Dysregulated type I interferon and inflammatory monocyte-macrophage responses cause lethal pneumonia in SARS-CoV-infected mice. Cell Host Microbe 19: 181–193. 26867177 10.1016/j.chom.2016.01.007 PMC4752723

[54] Luo, S., A. Vasbinder, J. M. Du‐Fay‐de‐Lavallaz, J. M. D. Gomez, T. Suboc, E. Anderson, A. Tekumulla, H. Shadid, H. Berlin, M. Pan, . 2022. Soluble urokinase plasminogen activator receptor and venous thromboembolism in COVID-19. J. Am. Heart Assoc. 11: e025198. 35924778 10.1161/JAHA.122.025198 PMC9683642

