# Supplementary material for: Targeting 2-Oxoglutarate–Dependent Dioxygenases Promotes Metabolic Reprogramming That Protects against Lethal SARS-CoV-2 Infection in the K18-hACE2 Transgenic Mouse Model
Source: Immunohorizons. 2023 Jul 7;7(7):528–42. doi: 10.4049/immunohorizons.2300048 (PMC10587500; doi:10.4049/immunohorizons.2300048)
Supplement: Supplemental Figures 1 (PDF) [file IH_2300048_Supplemental_1.pdf]

Figure S1

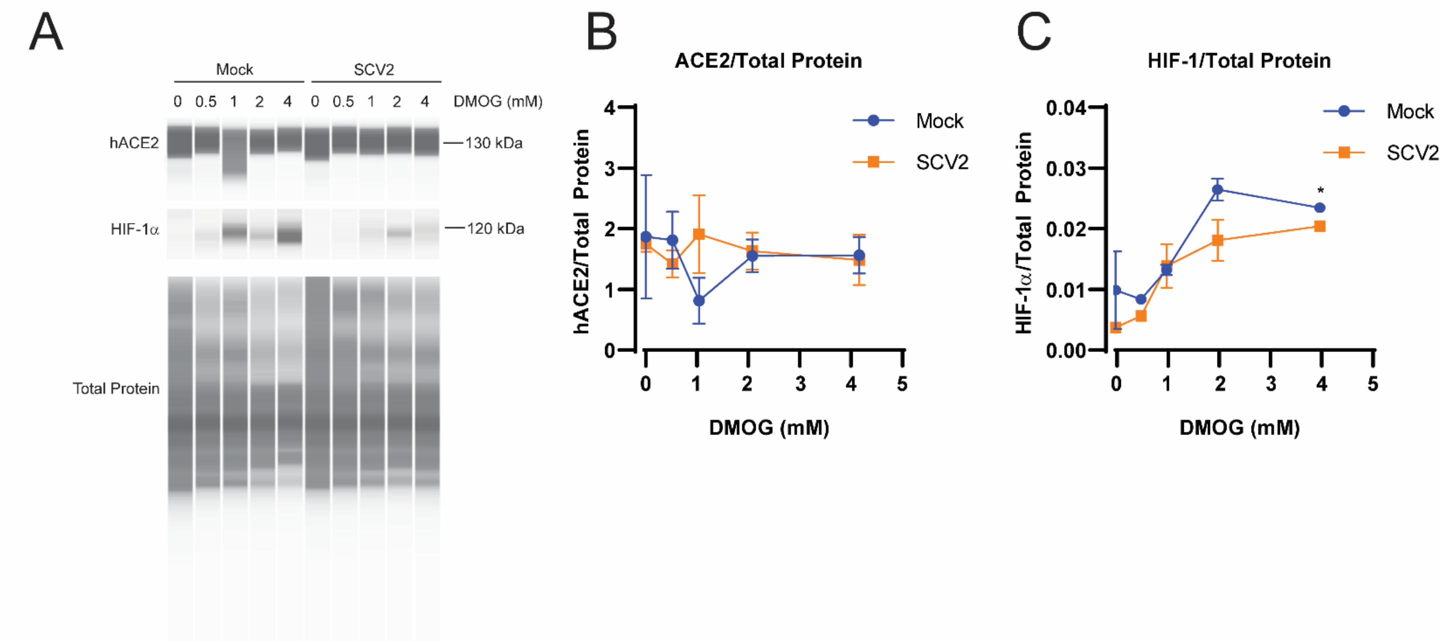

Figure S1: (A) Representative capillary based Western Blot of 3 separate repeats showing hACE2 and HIF-1α levels in hACE2-A549 cells 24 hours after infection with SARS-CoV-2 (MOI 0.1) with/without DMOG treatment. Accompanying graphs (B-C) show peak areas normalized to Total Protein Levels pooled from 3 separate experiments (mean +/- SEM, N=3). *$p<0.05$ indicates significance between mock and SCV2 infected mice using a student's *t*-test.

Figure S2

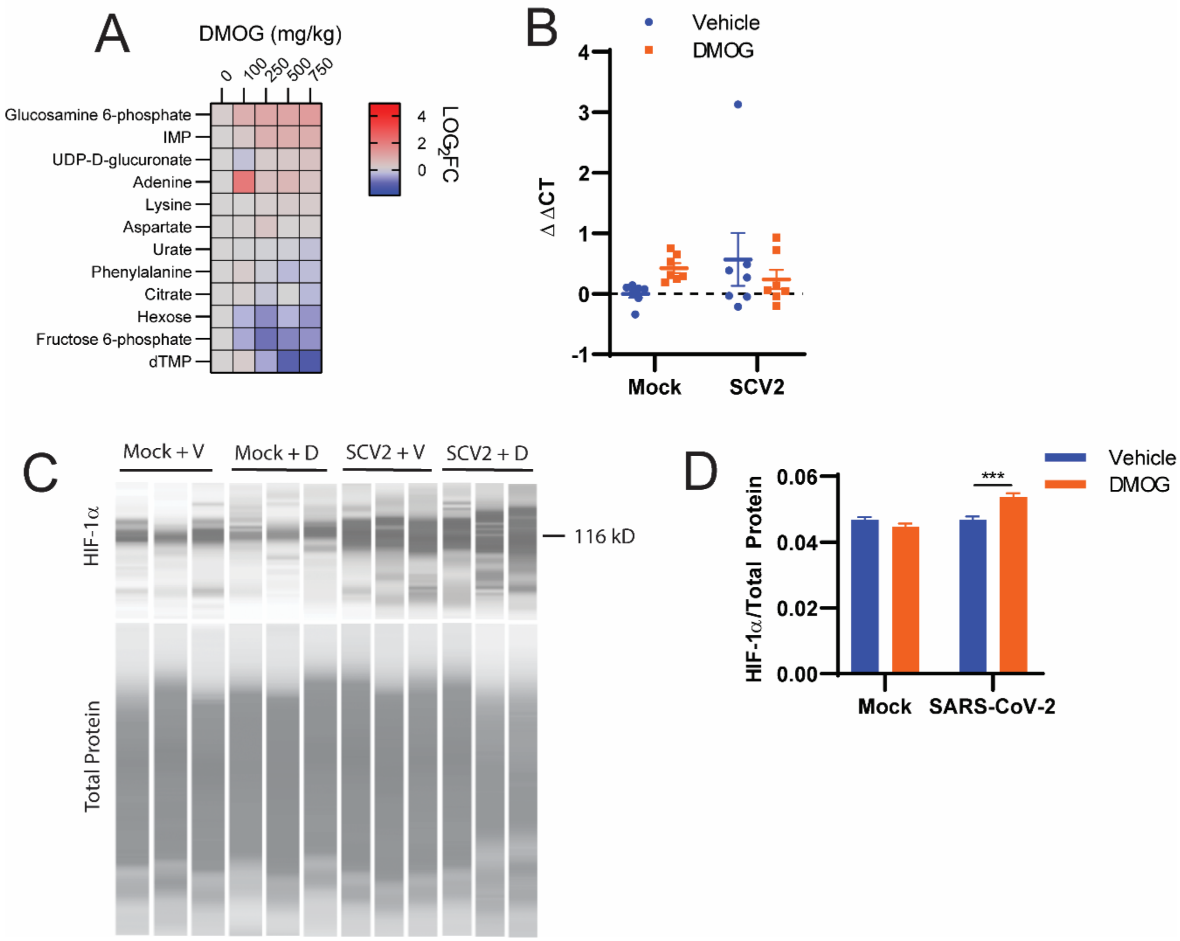

Figure S2: (A) Heatmap showing Log_2 (fold change) values of metabolites associated with central metabolism in mouse lungs 24 hours following treatment with increasing doses of DMOG or vehicle control (0 mg/kg dose). The metabolites presented shown are those that were found significant (P<0.05) relative to vehicle control at the 750 mg/kg dose (n=3) by students t-test. (B) Expression (ΔΔCT) values for hACE2 in the lungs of K18-hACE2 mice relative to uninfected controls. Data is shown as mean +/- SEM (N=7) pooled from 2 separate experimental repeats. (C) Representative microcapillary western blot projection of HIF-1α levels in mice with/without infection and vehicle or DMOG treatment (500 mg/kg) on day 5 post infection (3 individual mice shown for each treatment group). (D) Corresponding analysis of HIF-1α signal relative to total protein control for the studies shown in (C) (mean +/- SEM, N=5-7 per group pooled from 2 separate experimental repeats). ***p<0.001 indicates significance between SARS-CoV-2 infected with vehicle treatment relative to SARS-CoV-2 infection with DMOG treatment by 2-way ANOVA followed by Tukey's analysis for multiple comparisons.

Figure S3

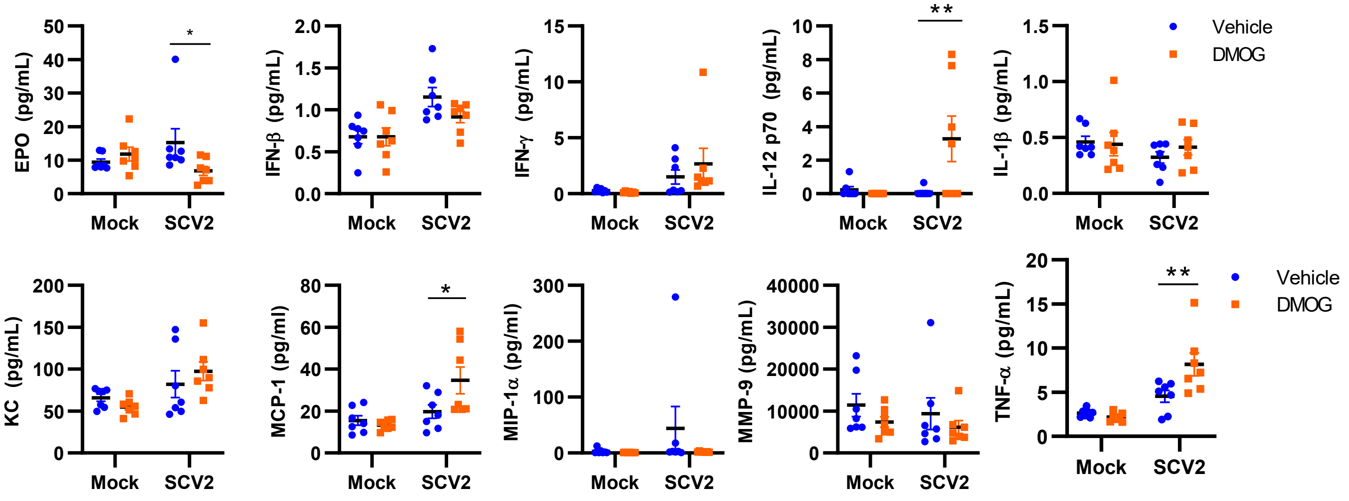

Figure S3: Inflammatory mediators in the serum of K18-hACE2 mice on day 5 post infection with or without DMOG treatment. Data is shown as mean +/- SEM (N=7, pooled from 2 separate experimental repeats). *p<0.05 indicates significance by 2-way ANOVA followed by Sidak correction for multiple comparisons.

Figure S4

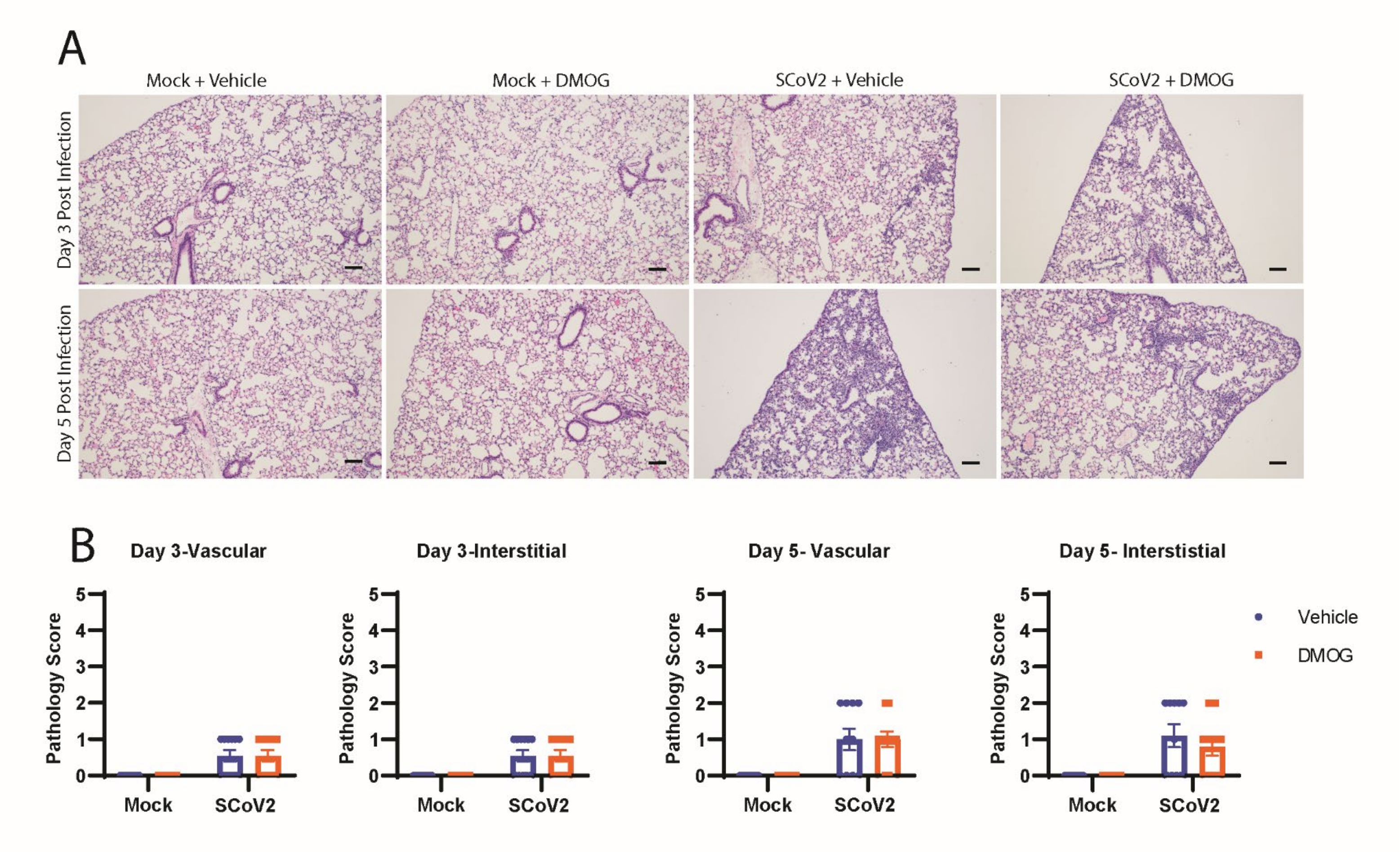

Figure S4: (A) Representative images of H&E-stained lung sections from infected and DMOG or vehicle treated mice on day 3 and 5 post infection. Black bar = 200 µm and images were acquired at 40X. (B) Associated pathological scores. Data is shown as mean score ± SEM (N=8-11 mice per group, combined from 2 separate experiments).
